# The Influence of a Table Tennis Physical Activity Program on the Gross Motor Development of Chinese Preschoolers of Different Sexes

**DOI:** 10.3390/ijerph18052627

**Published:** 2021-03-05

**Authors:** Ying Gu, Yong Chen, Jiameng Ma, Zhongyu Ren, Huaran Li, Hyunshik Kim

**Affiliations:** 1 College of Sports Science, Shenyang Normal University, Shenyang 110034, China; guying80@synu.edu.cn; 2 Department of Physical Education, Huaiyin Normal University, Huaian 223300, China; chenyong@hytc.edu.cn; 3 Faculty of Physical Education, Sendai University, Miyagi 9891693, Japan; jm-ma@sendai-u.ac.jp; 4 School of Physical Education, Southwest University, Chongqing 400715, China; renzhongyu@swu.edu.cn; 5 School of Foreign Languages, Shenyang Normal University, Shenyang 110034, China; lihuaran@synu.edu.cn

**Keywords:** table tennis, preschoolers, gross motor development, physical locomotion skills, object control skills

## Abstract

Gross motor locomotion is the basis of various sensory motor locomotion. Interventions helping preschoolers develop gross motor skills (GMS) could provide a solid foundation for complex motor skills. This study analyzed a table tennis physical activity program’s influence on preschoolers’ GMS development with 104 preschoolers (experimental group (EG): N = 52, 25 boys, 27 girls; control group (CG): N = 52, 25 boys, 27 girls). The EG conducted table tennis physical activities three times per week for 12 weeks. Preschoolers’ GMSs were assessed using the Test of Gross Motor Development (second edition). After 12 weeks, both the male and female EGs had significantly improved scores for GMS, locomotor subtest, gallop, hop, leap, slide, object control subtest, strike a stationary ball, stationary dribble, catch, overarm throw, and underhand roll (*p* < 0.05, *p* < 0.01, *p* < 0.001). The female EG also showed significant improvement for the run, horizontal jump, and catch in the post-test. Both the male and female EGs significantly outperformed the control group in their post-test scores for GMS, locomotor subtest, object control subtest, strike a stationary ball, overarm throw, and underhand roll (*p* < 0.05). The female EG also showed significant differences in slide scores (*p* < 0.05). Therefore, table tennis physical activities can promote preschoolers’ GMS development, especially object control skills. The research results provide an empirical basis for preschoolers’ physical education. Meanwhile, our findings have important implications for preschoolers’ GMS development and table tennis’ popularization in Chinese kindergartens.

## 1. Introduction

As an essential human skill, locomotion is the main instrument by which individuals interact with the external environment, and it plays a dual role in ensuring individuals’ survival and development [1]. The locomotion performed by gross muscles is called gross motor locomotion, and the development of gross muscles plays a crucial role in the process of motor development. Generally speaking, gross motor refers to locomotion in which the gross muscles of the trunk and limbs participate [2], and it includes the motor skills of physical locomotion and object control [3]. Children at the preschool and early education stage are the ideal learning subjects for developing gross motor skills [4]. For them, gross motor skills are the earliest-developed motor skills, and their development is beneficial to their physical health, mental cognition, and social adaptation [5,6,7]. Gross motor skills play an important role in preschoolers’ growth, development, and formation of an active lifestyle, and their sports psychology, sports interest, sports habits, sports cognition, and comprehensive sports quality are optimally developed through these skills’ promotion [8]. Furthermore, the development of gross motor skills can help preschoolers improve their confidence when participating in sports and lays a solid foundation for them to learn complex specialized skills in the future [9]. If such skills are not mastered by preschoolers, they may experience lifelong difficulties in learning motor skills in later life. Therefore, acquiring gross motor skills is crucial for their future development [10].

Gross motor skills are necessary to allow preschoolers to stably control their bodies and other objects while exploring the environment [11], and these skills need to be learned and strengthened through intervention [12], that is, planned exercise activities that are appropriate for preschoolers’ development [13]. Relevant studies have shown that participating in additional training of gross motor skills could significantly improve preschoolers’ performance in these skills, especially in physical locomotion and object control [14,15]. In China, there are few kindergartens that use intervention programs as physical education courses [16]; therefore, there is a lack of targeted exercises for preschool children’s large muscle motor skills. This leads to some preschoolers in the kindergarten physical education curriculum activities having gross motor skills that have not been fully developed. Further, there is scarce relevant literature on this topic [17]. Some related studies in China found that some sports programs, such as football [18], gymnastics [19], and badminton [20], had a promoting effect on the development of gross motor skills of preschool children. Furthermore, preschoolers’ physical activity was closely related to active family participation [21].

Table tennis is the most popular sport in China [22]. Because of the huge number of people who play table tennis, we focused on table tennis in our study. By learning table tennis, preschoolers can cultivate their ability to coordinate between their brain, hands, eyes, and feet, which is essential for the development of their speed, coordination, reflexes, endurance, flexibility, agility, and general physical quality. Furthermore, playing table tennis can also promote the development of preschoolers’ brains, especially their agility of thinking and stability of attention, as well as stimulate the development of their gross motor skills [23]. Previous studies have shown that table tennis could help improve preschoolers’ gross motor skills [24]. Most of these focused on primary school students and teenagers, while only a few studies constituted table tennis-related interventions on preschoolers’ gross motor skills. Therefore, this research takes the table tennis project in Chinese kindergartens as the starting point to explore the influence of table tennis on preschoolers’ GMS, and by comparing the difference between experimental group and control group on children’s gross motor skills, the pros and cons of the two programs are analyzed. In the course of their research, Foweather [25] found that boys and girls have different levels of gross motor development in the intervention process. In order to understand the impact on boys and girls during the intervention process, we separately analyzed the boys and girls and conducted empirical explorations to promote the development of children’s gross movements.

The Test of Gross Motor Development, second edition (TGMD-2) [3] attaches importance to the development of preschoolers’ gross motor skills. It is rich in content and easy to perform. Since the TGMD-2 has been widely used in the United States [3], Australia [26], Brazil [27], Belgium [28], and other countries, its reliability and validity can be confirmed, and Chinese scholars believe that it is also suitable for China [29,30]. Therefore, in this study, the TGMD-2 was selected as the test tool, 3- to 6-year-old preschoolers were taken as the study subjects, and a table tennis program was used to improve preschoolers’ gross motor ability and lay a solid foundation for cultivating their lifelong physical exercise awareness. The study aimed to formulate appropriate table tennis courses and promote the playing of table tennis in kindergartens by studying the influence of table tennis physical activity programs on the gross motor skills of Chinese preschoolers.

## 2. Materials and Methods

### 2.1. Study Objects

The samples of this study were from five-star kindergartens (the highest level of kindergartens in China) in Liaoning Province, China. This samples were 104 children aged 3–6 years (50 boys, 54 girls). Boys and girls were randomly divided into two groups: one group was an experimental group with 52 children (25 boys and 27 girls), the other group was a control group with 52 children (25 boys and 27 girls). The study participants’ basic information is shown in Table 1, Table 2 and Table 3. The study was conducted with the consent of their parents or guardians and approved by the Human Ethics Research Committee of the School of Physical Education and Sports Science, Shenyang Normal University (SYNU19-09). In this study, homogeneity tests were performed on participants.

The results of the homogeneity test showed no significant difference in the basic characteristics of the total, male, and female samples between the experimental group and control group (*p* > 0.05). Therefore, all groups had homogeneity.

### 2.2. Study Instrument

The TGMD-2 was used to measure and evaluate preschool children’s level of gross motor development [3]. Their physical locomotion ability and object control ability were both tested. The test of physical locomotion ability includes a test of six actions—running, galloping, hopping, horizontal jumping, leaping, and sliding—while the test of object control ability also consists of six actions—striking a stationary ball, catching, kicking, stationary dribbling, overhand throwing, and underhand rolling. These standards could be used to measure whether children’s trunk and limbs were coordinated, whether the formation of their gross motor skills was correct, and whether a natural and flexible motor skill pattern could be achieved when they were completing a certain motor skill. In the pre-test and post-test, each action in the TGMD-2 was tested twice to get the best result. Scores were given according to 3–5 criteria, for 1 point each. If a child failed to meet any criterion, 0 points would be given. The original score for physical locomotion was 48, while the original score for object control was also 48.

### 2.3. Study Procedures

**Figure 1:**
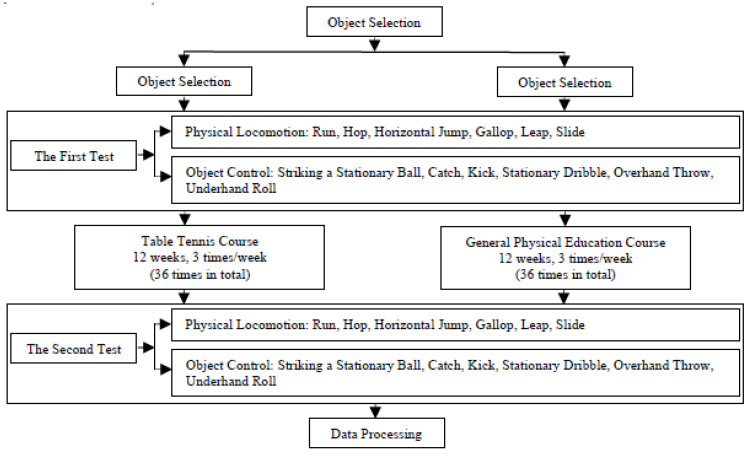
Study procedures.

Based on the theoretical system of children’s table tennis training [31,32,33,34] and basic table tennis motor skills [35], this study constructed the training of physical locomotion skills and object control skills [3] according to the TGMD-2 and table tennis courses designed to develop preschoolers’ gross motor skills. The experiment began in October 2019, and classes occurred three times a week, for 50 min each time, for a total of 12 weeks. Classes for the experimental group and control group were completed together with two highly trained physical education teachers. The experimental group carried out special table tennis training according to the design content (shown in Table 4). The control group carried out regular physical education courses (such as sports games and outdoor activities) formulated by the kindergarten according to the index system content of China’s “Guidelines for Kindergarten Education (Trial) [36]” and “Guidelines for the Development of Children Aged 3–6” [37]. The two testers who tested the children before and after the experiment had a full understanding of the TGMD-2 and rich field experience in sports tests for children. The test environment and tools had also been checked to ensure that the test could be completed successfully (shown in Figure 1).

### 2.4. Mathematical Statistics

In the pre-test, an independent sample T-test was conducted on the basic characteristics of the experimental group and control group. In terms of age, height, weight, and basic motor skills, there was no statistical difference between the groups (*p* > 0.05). Therefore, the experimental group and control group were considered homogeneous. In the post-test, the basic data of the experimental group and control group were analyzed for mean and standard deviation. An independent sample T-test was conducted between the two groups. Additionally, a paired sample T-test was performed on the pre-post data in the groups, while an independent sample T-test was conducted to ascertain the differences between the experimental group and control group in the pre-test and post-test. The T-test indicated that the differences had statistical significance (*p* < 0.05). In this study, SPSS WIN 25.0 was used for statistical analysis.

## 3. Results

### 3.1. Results of Male Preschoolers

As shown in Table 5, the gross motor skills score of the male experimental group was 62.7 ± 11.6 in the pre-test and 75.4 ± 11.6 in the post-test. Thus, the post-test score increased by 12.6 ± 2.86, which showed a significant difference (*p* < 0.001). The gross motor skills score of the male control group was 61.4 ± 1.20 in the pre-test and 66.8 ± 12.2 in the post-test. Thus, the post-test score increased by 5.40 ± 3.14, which showed no significant difference. In the post-test, the gross motor skills score of the experimental group was 75.4 ± 11.6, while that of the control group was 66.8 ± 12.2, which corresponded to a significant difference (*p* < 0.05). In sum, there was a significant difference in the gross motor skills scores of the male experimental group and control group between the pre-test and post-test (*p* < 0.001).

Regarding locomotor subtest skills, as shown in Table 6, in the male experimental group, the scores for the locomotor subtest, gallop, hop, leap, and slide showed significant differences between the pre-test and post-test (*p* < 0.05, *p* < 0.01), while there was no significant difference between them in the run and horizontal jumps in the pre-test and post-test. In the control group, the locomotor subtest score was significantly different between the pre-test and post-test (*p* < 0.05), while in the run, gallop, hop, leap, horizontal jump, and slide, there were no significant differences between them in the pre-test and post-test. In the post-test, the locomotor subtest score of the experimental group was significantly different from that of the control group (*p* < 0.05). There was also no significant difference in the run, gallop, hop, leap, horizontal jump, and slide between the experimental group and control group. The scores for the locomotor subtest, gallop, horizontal jump, and slide were significantly different between the experimental group and control group (*p* < 0.05, *p* < 0.01, *p* < 0.001), while there was no significant difference in the run, hop, leap, and horizontal jumps between the experimental group and control group.

Regarding the object control subtest skills, as shown in Table 6, in the male experimental group, there were significant differences in the scores for the object control subtest, strike a stationary ball, stationary dribble, catch, overarm throw, and underhand roll (*p* < 0.05, *p* < 0.01, *p* < 0.001) between the pre-test and post-test. There was no significant difference in kick in the experimental group between the pre-test and post-test, while in the control group, there was no significant difference in the scores for the object control subtest, strike a stationary ball, stationary dribble, catch, kick, overarm throw, and underhand roll between the pre-test and post-test. Compared with the control group, the experimental group presented significant differences in their scores for the object control subtest, strike a stationary ball, overarm throw, and underhand roll (*p* < 0.05), but no significant difference in the stationary dribble, catch, and kick in the post-test. Hence, there were significant differences in the scores for the object control subtest, strike a stationary ball, stationary dribble, catch, overarm throw, and underhand roll (*p* < 0.01, *p* < 0.001), but no significant difference in the kick between the experimental group and control group in the pre-test and post-test.

### 3.2. Results of Female Preschoolers

As shown in Table 7, the gross motor skills score of the female experimental group was 59.48 ± 10.52 in the pre-test and 72.41 ± 10.00 in the post-test. Thus, the post-test score increased by 12.93 ± 3.30, which showed a significant difference (*p* < 0.001). The gross motor skills score of the female control group was 60.93 ± 10.65 in the pre-test and 66.07 ± 10.11 in the post-test. Thus, the post-test score increased by 5.15 ± 2.20, which showed no significant difference. In the post-test, the gross motor skills score of the experimental group was 72.41 ± 10.00, while that of the control group was 66.07 ± 10.11, which demonstrated a significant difference (*p* < 0.05). To summarize, there was a significant difference in gross motor skills scores between the female experimental group and control group in the pre-test and post-test (*p* < 0.001).

Regarding locomotor subtest skills, as shown in Table 8, in the female experimental group, the score of the locomotor subtest, run, gallop, hop, leap, horizontal jump, and slide showed significant differences in the pre-test and post-test (*p* < 0.05, *p* < 0.01, *p* < 0.001). In the control group, the score for the locomotor subtest, run, gallop, hop, leap, horizontal jump, and slide showed no significant difference between the pre-test and post-test. Compared with the control group, the experimental group presented significant differences in their score for the slide (*p* < 0.05) but no difference in their scores for the locomotor subtest, run, gallop, hop, leap, and horizontal jump in the post-test. The score for the locomotor subtest, hop, leap, and horizontal jump between the experimental group and control group were significantly different in the pre-test and post-test (*p* < 0.05, *p* < 0.01, *p* < 0.001), while there were no significant differences in the run, gallop, and horizontal jump between the experimental group and control group in the pre-test and post-test.

Regarding object control subtest skills, as shown in Table 8, in the female experimental group, there were significant differences in their scores for the object control subtest, strike a stationary ball, stationary dribble, catch, kick overarm throw, and underhand roll (*p* < 0.05, *p* < 0.01, *p* < 0.001) between the pre-test and post-test. Moreover, in the control group, there was no significant difference in their scores for the object control subtest, strike a stationary ball, stationary dribble, catch, kick, overarm throw, and underhand roll between the pre-test and post-test. Compared with the control group, the experimental group presented significant differences in their scores for the object control subtest, strike a stationary ball, overarm throw, and underhand roll (*p* < 0.05), but no significant difference in the stationary dribble, catch, and kick in the post-test. Hence, there were significant differences in the object control subtest score, strike a stationary ball, stationary dribble, catch, overarm throw, and underhand roll (*p* < 0.001), but no significant difference in kick between the experimental group and control group in the pre-test and post-test.

## 4. Discussion

The purpose of this study was to analyze the influence of table tennis on the gross motor development of preschoolers of different sexes in China through a table tennis physical activity program. A total of 104 preschoolers were selected as study subjects, and the TGMD-2 [3] was used as the test tool. The results were as follows:

In the male and female experimental groups, the total scores for gross motor skills, physical locomotion, and object control improved significantly (*p* < 0.01), while no significant change occurred in the control group between the pre-test and post-test. The results showed that the table tennis program could effectively improve preschoolers’ gross motor skills, including physical locomotion skills and object control skills. In their study of 78 preschoolers, Šalaj et al. [40] found that children who participated in sports activities had better performance in a motor skills test, which is consistent with the results of our study. Robinson et al. [41] conducted exercise intervention on 113 preschoolers in the United States, testing them with the TGMD-2, and the research results showed that the gross motor skills of preschoolers could be significantly improved. Mostafavi et al. [42] conducted a Sports, Play, and Active Recreation for Kids (SPARK) course for eight weeks with 90 children aged 4–6 years in Iran, and their results showed that SPARK courses had higher efficacy in promoting basic motor skills than common physical education courses, thus also confirming the results of our study. Further, the study by Ping et al. [17] on the influence of a physical activity program on children’s gross motor development suggests that a table tennis program could significantly improve children’s gross motor ability. To some extent, it could also improve children’s physique level and promote their physical health. Some other previous literature has presented similar results, including the study by Shengkou et al. [43] on the physical activity exercises of 289 children in China, which showed that physical activity programs could promote the development of children’s gross motor skills.

Compared with the control group, the experimental group of male children showed significant improvement in total score for gross motor skills, physical locomotion, and object control (*p* < 0.05), while the experimental group of female children also presented significant improvement in total score for gross motor skills and object control (*p* < 0.05). The study conducted by Jianlong et al. [44] on 2136 preschoolers in China showed that children in the experimental group outperformed those in the control group in terms of physical locomotion skills and object control skills. Šalaj et al. [40] also believed that children who participated in organized exercise programs had better performance in motor development than those who did not. In the current study, the post-test results of the experimental group and control group were similar to those of Šalaj et al. [40], but differed in that the total score of female children’s physical locomotion skills did not show a significant difference between the pre-test and post-test, perhaps because of the different test content. Jianlong et al. [44] used a three-stage modern physical teaching method, while Šalaj et al. [40] used a mix of various sports methods.

In the current study, the male children in the experimental group presented significant improvement in the slide, stationary dribble, striking a stationary ball, overhand throw, underhand roll, leap, hop, horizontal jump, and catch (*p* < 0.05, *p* < 0.01), while the female children presented significant improvement in the slide, stationary dribble, overhand throw, and underhand roll in the post-test (*p* < 0.05). This showed that table tennis could improve the skills of both male and female children in the slide, stationary dribble, overhand throw, and underhand roll, while the non-development of other motor skills might have been caused by various factors, such as individual growth, living habits, different family education ideas about different genders, and the like. In the study by Foweather et al. [25] on 99 children, the male children were found to be more active than the female children, which could support this idea. Therefore, more programs showed significant changes for male children than for female children in the post-tests.

Brian A. et al. [45] conducted an eight-week physical activity intervention study on preschoolers in the United States, using the TGMD-2 test, and the results of the study showed positive changes in the object manipulation ability of preschoolers. Honglu’s [46] study on Chinese children’s ball-based physical activity showed that ball games could promote their basic motor skills and improve the development of their physical locomotion and object control ability. Furthermore, the influence of ball games on the development of object control skills was greater than that on the development of physical locomotion skills. Our test showed the same influence from balls. In our study, besides the slide, there was no significant difference in the physical locomotion or other motor skills of female children, perhaps because of the locomotive characteristics of the table tennis activity itself.

In Yuanyuan’s [47] study of 177 children aged 5–6 years, the children’s kicking ability was not improved effectively. Since her study was similar to ours, this may have been because of the lack of “kicking” in both experiments, with children seldom using such actions during the experiment. Šalaj et al. [40] pointed out that children who participated in multiple sports outperformed those who did not (or who only participated in a single sport) and suggested that multi-sport participation could be recommended as the best form of exercise for preschoolers. In our study, there was no significant difference in the stride, gallop, or kick of male children and the stride, leap, hop, horizontal jump, gallop, catch, and kick between the experimental group and control group in the post-test. This showed that the influence of certain skills on gross motor development was limited by the characteristics of certain sports. Therefore, we should design diverse sports programs and comprehensive development methods to promote the development of children’s gross motor skills. Choosing appropriate sports programs [48] could effectively promote preschoolers’ motor development [49].

Some limitations need to be considered in the interpretation of our study findings. First, due to the characteristics of table tennis, the results of this study on gross motor development might differ from those of other sports. Second, in view of single sports’ technical limitations, sports programs for children with diverse educational outcomes should be designed to achieve the goal of all-round development of children’s skills. Third, this study did not consider the influence of children’s personal qualities, their family’s economic and cultural levels, or their parents’ educational views. Therefore, the representativeness of the sample was limited. Fourth, the control group was also limited by other uncontrollable factors besides the physical education course, such as after-school play, extracurricular activities, etc. Those factors might also have led to differences between our study results and those of others. Nevertheless, the study results provide powerful support for the development of gross motor skills among Chinese preschoolers. Since this study focused on Chinese preschoolers, the results are of great significance for Chinese preschool education institutions in designing better physical activity training courses and more effective intervention programs for preschool children in the future.

## 5. Conclusions

In the male sample, there was significant improvement in the scores for gross motor skills, locomotor subtest, gallop, hop, leap, slide, object control subtest, strike a stationary ball, stationary dribble, catch, overarm throw, and underhand roll (*p* < 0.05, *p* < 0.01, *p* < 0.001) in the experimental group between the pre-test and post-test. Compared with the control group, the experimental group presented significant differences in their scores for gross motor skills, locomotor subtest, object control subtest, strike a stationary ball, overarm throw, and underhand roll (*p* < 0.05). In the female sample, there was significant improvement in their scores for gross motor skills, locomotor subtest, run, gallop, hop, leap, horizontal jump, slide, object control subtest, strike a stationary ball, stationary dribble, catch, kick, overarm throw, and underhand roll (*p* < 0.05, *p* < 0.01, *p* < 0.001) in the experimental group between the pre-test and post-test. Compared with the control group, the experimental group presented significant differences in their scores for gross motor skills, slide, object control subtest, strike a stationary ball, overarm throw, and underhand roll (*p* < 0.05). The study confirms that table tennis can effectively improve the gross motor skills of preschoolers aged 3–6 years, especially their object control skills, and it also provides empirical evidence for preschoolers’ physical education. Therefore, this study is of great significance for educational institutions when designing physical activity training courses for preschoolers and in the study of children’s physical development.

## Figures and Tables

**Table 1:** Total subject variance homogeneity test.

Variable	EG (n = 52)	CG (n = 52)	Levene’s Test		T-Test	
	M ± SD		F	*p*	T	*p*
Age (month)	55.190 ± 8.702	55.190 ± 8.997	0.068	0.770	0.000	1.000
Height (cm)	107.171 ± 7.649	107.235 ± 7.717	0.099	0.754	−0.042	0.966
Weight (kg)	17.504 ± 3.432	17.529 ± 2.845	0.661	0.418	−0.040	0.968

CG: control group; EG: experimental group; M: mean.

**Table 2:** Male subject variance homogeneity test.

Variable	EG (n = 25)	CG (n = 25)	Levene’s Test		T-Test	
	M ± SD		F	*p*	T	*p*
Age (month)	54.760 ± 8.927	54.880 ± 8.983	0.009	0.923	−0.047	0.962
Height (cm)	107.280 ± 7.431	107.868 ± 6.956	0.000	0.999	−0.298	0.774
Weight (kg)	18.140 ± 3.462	17.596 ± 2.554	0.778	0.382	0.632	0.530

CG: control group; EG: experimental group; M: mean.

**Table 3:** Female subject variance homogeneity test.

Variable	EG (n = 27)	CG (n = 27)	Levene’s Test		T-Test	
	M ± SD		F	*p*	T	*p*
Age (month)	55.590 ± 8.639	55.480 ± 9.171	0.254	0.617	0.046	0.964
Height (cm)	107.070 ± 7.986	106.648 ± 8.451	0.139	0.711	0.189	0.851
Weight (kg)	16.915 ± 3.359	17.467 ± 3.139	0.015	0.904	−0.624	0.536

CG: control group; EG: experimental group; M: mean.

**Table 4:** Table tennis course content.

Module	Theme	Content	Function
**Narrative Module**	In this module, characters are shaped through certain story plots (or scripts) so as to express the main idea of the designed activities [38]. The story-telling method is also used to guide preschoolers into the theme.	Each activity of the module has its own anthropomorphic name (e.g., Little Bear Lifting Ball, Monkey Picking Fruit, Little Swallows Flying Together, Ant Moving House, Squirrel Picking Fruit, Little Snake Racing, Cat Fishing, Rabbit Shooting, Chicken Pecking Rice, Bird Catching Bug, etc. [39].	To stimulate preschoolers’ curiosity, attention, and interest in learning.
**Game Module**	Connected to the narrative module, the game module aims to stimulate preschoolers’ enthusiasm for games through previously imported stories.	For instance, in the activity of Ants Moving House, preschoolers can imitate ants moving by moving ping-pong balls. By doing so, each child can play a role in the activity in single or multi-player cooperation.	To improve preschoolers’ skills, guide them to experience the sense of achievement and honor, enjoy the pleasure of success and victory, and enhance their self-confidence.
**Skills Module**	Get to know the ball	Pick up the ball, throw the ball, change the ball, throw the ball overhand, pass the ball, and catch the ball.	To cultivate preschoolers’ ability to coordinate between brain, hands, and eyes, and enhance their sensory integration and coordination.
	Perceive the ball	Dribble the stationary ball, dribble the rebounded ball, toss the ball, and bump the ball, cover the ball, and hit the ball against the wall.	To exercise preschoolers’ motor ability and sense of direction and improve their body balance.
	Imitation	Mimic locomotion: grip, swing, forehand swing, and backhand swing, flat serve. Mimic footwork: single step, slide step, stride step, cross step, parallel step, single-foot jump, double-foot jump.	To form preschoolers’ muscle memory and consolidate their motor skills by imitating locomotion of the upper and lower limbs.
	Mastering skills	Forehand flat shot, forehand attack, backhand attack, forehand alternate attack, footwork movement attack, forehand rallies, backhand rallies, and simple tactical games.	To exercise the flexibility, control, and coordination of preschoolers’ limbs, cultivate their ability to cooperate with each other, and guide them to experience the happiness brought by table tennis and the sense of achievement after hitting the ball successfully.
**Summary Module**	Relaxing	Stretch each other slowly, jogging, deep breathing, massage.	To relax the body in order to prevent injury, improve retraining ability, and promote health.
	Summarizing and communicating	Interact with preschoolers in plain child language to find out whether they grasp and understand the content of the course.	To stimulate preschoolers’ gumption, enhance their self-confidence, and promote their interest in the course. To make them look forward to and actively participate in the next class.

**Table 5:** Comparison of total gross motor skills pre- and post-test scores between the male preschooler EG and CG.

Object	Group	Pre	Post	Pre–Post Difference	T
		M ± SD			
Total GMS Score	EG (n = 25)	62.7 ± 11.6	75.4 ± 11.6	12.6 ± 2.86	−3.86 ***
	CG (n = 25)	61.4 ± 1.20	66.8 ± 12.2	5.40 ± 3.14	−1.58
	T	0.40	2.55 *	8.54 ***	

CG: control group; EG: experimental group; GMS: gross motor skills. * *p* < 0.05, *** *p* < 0.001.

**Table 6:** Comparison of locomotor subtest skills and object control subtest skills between male EG and CG pre- and post-test intervention.

Object		Group	Pre	Post	Pre–Post Difference	T
			M ± SD			
Locomotor Subtest Skills	Locomotor subtest score	EG (n = 25)	34.1 ± 6.10	39.8 ± 6.11	5.72 ± 2.01	−3.33 **
		CG (n = 25)	32.1 ± 5.81	35.6 ± 5.70	3.58 ± 2.69	−2.19
		T	1.19	2.49 *	7.70 ***	
	Run	EG (n = 25)	6.76 ± 1.01	7.42 ± 1.01	0.48 ± 0.65	−1.69
		CG (n = 25)	6.32 ± 1.41	6.84 ± 1.07	0.52 ± 0.99	−1.47
		T	1.27	1.36	−0.177	
	Gallop	EG (n = 25)	4.88 ± 1.20	5.80 ± 1.32	0.92 ± 0.64	−2.57 *
		CG (n = 25)	4.76 ± 1.23	5.12 ± 1.05	0.36 ± 0.49	−1.11
		T	0.35	2.01	3.47 **	
	Hop	EG (n = 25)	5.84 ± 2.17	7.24 ± 2.39	1.40 ± 1.04	−2.17 *
		CG (n = 25)	5.32 ± 2.41	6.28 ± 2.21	0.96 ± 1.10	−1.47
		T	0.80	1.48	1.45	
	Leap	EG (n = 25)	5.04 ± 1.06	5.72 ± 0.981	0.68 ± 0.63	−2.46 *
		CG (n = 25)	5.00 ± 1.04	5.40 ± 0.957	0.40 ± 0.66	−1.41
		T	0.14	1.22	1.56	
	Horizontal jump	EG (n = 25)	5.80 ± 2.27	6.60 ± 1.83	0.80 ± 0.71	−1.37
		CG (n = 25)	5.28 ± 1.70	5.88 ± 1.59	0.60 ± 0.76	−1.29
		T	0.92	1.49	0.961	
	Slide	EG (n = 25)	5.76 ± 2.01	7.20 ± 1.50	1.44 ± 1.16	−2.88 **
		CG (n = 25)	5.40 ± 1.56	6.12 ± 1.76	0.72 ± 0.74	−1.45
		T	0.68	2.33	2.62 *	
Object Control Subtest Skills	Object control subtest score	EG (n = 25)	28.60 ± 6.56	35.60 ± 6.68	6.92 ± 2.08	−3.70 **
		CG (n = 25)	29.32 ± 7.02	31.16 ± 7.19	1.84 ± 1.38	−0.92
		T	−0.35	2.24 *	10.19 ***	
	Strike a stationary ball	EG (n = 25)	5.64 ± 1.91	7.12 ± 1.83	1.84 ± 0.77	−2.79 **
		CG (n = 25)	5.60 ± 1.89	5.76 ± 1.86	0.16 ± 0.37	−0.30
		T	0.07	2.61 *	7.71 ***	
	Stationary dribble	EG (n = 25)	3.16 ± 1.80	4.60 ± 1.73	1.44 ± 1.08	−2.89 **
		CG (n = 25)	3.16 ± 1.77	3.84 ± 1.99	0.68 ± 0.75	−1.28
		T	0.000	1.44	2.89 **	
	Catch	EG (n = 25)	4.08 ± 1.26	4.84 ± 1.34	0.76 ± 0.83	−2.07 *
		CG (n = 25)	4.20 ± 1.44	4.28 ± 1.93	0.08 ± 0.28	−0.20
		T	−0.31	1.43	3.88 ***	
	Kick	EG (n = 25)	4.29 ± 1.58	5.64 ± 1.55	0.72 ± 0.61	−1.63
		CG (n = 25)	5.12 ± 1.74	5.68 ± 1.80	0.56 ± 0.65	−1.12
		T	−0.43	−0.08	0.89	
	Overarm throw	EG (n = 25)	5.48 ± 1.23	6.80 ± 1.23	1.32 ± 0.85	−3.80 ***
		CG (n = 25)	5.56 ± 1.42	5.84 ± 1.43	0.28 ± 0.46	0.70
		T	−0.21	2.55 *	5.37 ***	
	Underhand roll	EG (n = 25)	5.44 ± 1.23	6.56 ± 1.39	1.12 ± 0.93	−3.02 **
		CG (n = 25)	5.68 ± 1.35	5.76 ± 1.33	0.08 ± 0.28	−0.21
		T	−0.66	2.08 *	5.37 ***	

CG: control group; EG: experimental group; * *p* < 0.05, ** *p* < 0.01, *** *p* < 0.001.

**Table 7:** Comparison of total gross motor skills pre- and post-test scores between the female preschooler EG and CG.

Object	Group	Pre	Post	Pre-Post Difference	T
		M ± SD			
Total GMS score	EG (n = 27)	59.48 ± 10.52	72.41 ± 10.00	12.93 ± 3.30	−4.63 ***
	CG (n = 27)	60.93 ± 10.65	66.07 ± 10.11	5.15 ± 2.20	−1.82
	T	−0.501	2.31 *	10.19 ***	

CG: control group; EG: experimental group; GMS: gross motor skills. * *p* < 0.05, *** *p* < 0.001.

**Table 8:** Comparison of locomotor subtest skills and object control subtest skills between female EG and CG pre- and post-intervention.

Object		Group	Pre	Post	Pre–Post Difference	T
			M ± SD			
Locomotor subtest skills	Locomotor subtest score	EG (n = 27)	33.48 ± 5.83	39.11 ± 4.77	5.63 ± 2.62	−3.88 ***
		CG (n = 27)	33.74 ± 6.24	36.70 ± 5.51	2.96 ± 1.63	−1.85
		T	−0.16	1.72	4.49 ***	
	Run	EG (n = 27)	6.11 ± 1.09	6.70 ± 1.03	0.59 ± 0.80	−2.06 *
		CG (n = 27)	6.44 ± 1.12	6.85 ± 1.23	0.41 ± 0.69	−1.27
		T	−1.11	−0.48	0.91	
	Gallop	EG (n = 27)	5.04 ± 1.13	5.74 ± 1.06	0.70 ± 0.54	−2.37 *
		CG (n = 27)	4.93 ± 1.52	5.44 ± 1.25	0.52 ± 0.58	−1.37
		T	0.31	0.94	1.21	
	Hop	EG (n = 27)	6.22 ± 2.62	7.59 ± 2.02	1.37 ± 1.15	−2.15 *
		CG (n = 27)	6.19 ± 2.43	6.82 ± 2.06	0.63 ± 0.79	−1.03
		T	0.05	1.40	2.76 **	
	Leap	EG (n = 27)	4.63 ± 1.01	5.41 ± 1.01	0.78 ± 0.64	−2.84 **
		CG (n = 27)	4.70 ± 0.95	5.04 ± 1.19	0.33 ± 0.62	−1.14
		T	−0.28	1.23	2.59 *	
	Horizontal jump	EG (n = 27)	5.74 ± 1.63	6.59 ± 1.42	0.85 ± 1.03	−2.05 *
		CG (n = 27)	5.96 ± 2.05	6.44 ± 1.67	0.48 ± 0.64	−0.95
		T	−0.44	0.35	1.59	
	Slide	EG (n = 27)	5.74 ± 1.58	7.07 ± 1.21	1.33 ± 1.33	−3.48 **
		CG (n = 27)	5.52 ± 1.85	6.11 ± 1.65	0.59 ± 0.75	−1.24
		T	0.48	2.45 *	2.52 *	
Object control subtest skills	Object control subtest score	EG (n = 27)	26.00 ± 6.36	33.30 ± 6.32	7.30 ± 2.09	−4.23 ***
		CG (n = 27)	27.19 ± 5.98	29.37 ± 5.81	2.19 ± 1.57	−1.36
		T	−0.71	2.38 *	10.16 ***	
	Strike a stationary ball	EG (n = 27)	4.37 ± 2.15	6.04 ± 1.79	1.67 ± 0.83	−3.10 **
		CG (n = 27)	4.70 ± 2.16	5.00 ± 1.98	0.30 ± 0.47	−0.53
		T	−0.57	2.02 *	7.47 ***	
	Stationary dribble	EG (n = 27)	2.82 ± 2.00	4.52 ± 1.95	1.70 ± 0.95	−3.17 **
		CG (n = 27)	3.22 ± 1.99	3.78 ± 2.06	0.56 ± 0.64	−1.01
		T	−0.75	1.36	5.19 ***	
	Catch	EG (n = 27)	4.00 ± 1.30	4.78 ± 1.37	0.78 ± 0.58	−2.14 *
		CG (n = 27)	4.15 ± 1.20	4.30 ± 1.14	0.15 ± 0.36	−0.47
		T	−0.44	1.41	4.80 ***	
	Kick	EG (n = 27)	3.89 ± 1.37	4.70 ± 1.51	0.81 ± 0.79	−2.08 *
		CG (n = 27)	4.00 ± 1.59	4.52 ± 1.70	0.52 ± 0.80	−1.16
		T	−0.28	0.42	1.37	
	Overarm throw	EG (n = 27)	5.37 ± 1.45	6.56 ± 1.16	1.19 ± 0.83	−3.78 ***
		CG (n = 27)	5.44 ± 1.22	5.85 ± 1.03	0.41 ± 0.64	−1.33
		T	−0.23	2.37 *	3.85 ***	
	Underhand roll	EG (n = 27)	5.56 ± 1.31	6.70 ± 1.44	1.15 ± 0.82	−3.07 **
		CG (n = 27)	5.67 ± 1.14	5.93 ± 1.39	0.26 ± 0.53	−0.75
		T	−0.33	2.03 *	4.75 ***	

CG: control group; EG: experimental group; * *p* < 0.05, ** *p* < 0.01, *** *p* < 0.001.

## Data Availability

Data provided in this study are available upon request by the corresponding author. The data were not made public because basic information on children was designed to be tested.
